# Nurses’ perceptions of continuing professional development: a qualitative study

**DOI:** 10.1186/s12912-022-00940-z

**Published:** 2022-06-23

**Authors:** Xiaoyan Yu, Yi Huang, Yu Liu

**Affiliations:** Department of Nursing, Tongji Hospital, Tongji Medical College, Huazhong University of Science and Technology, No. 1095 Jiefang Avenue, Wuhan, 430030 China

**Keywords:** Continuing professional development, Nursing, Qualitative study

## Abstract

**Background:**

Continuing professional development is regarded as one of the important approaches to maintaining skills and motivation for work. However, there is a lack of qualitative studies to explore Chinese nurses’ continuing professional development. The study aims to explore Chinese nurses’ perceptions of continuing professional development and challenges they face.

**Methods:**

The study was conducted in a tertiary hospital located in the central region of China from July to August 2020. Purposive sampling was used to recruit 14 nurses and face to face semi-structured interviews were conducted from July to August 2020. Then the recorded data were analysed and collated according to the thematic analysis. This study followed the consolidated criteria for reporting qualitative research (COREQ).

**Results:**

Four themes were extracted: improving specialty ability; different development phases; the importance of personal effort; the obstacle of work-family conflict.

**Conclusions:**

This study contributed to our understandings of nurses’ continuing professional development. Nurses held a positive attitude towards continuing professional development and they faced challenges in the meantime. Special attention and targeted supports should be provided to promote the continuing professional development of nursing staff.

**Supplementary Information:**

The online version contains supplementary material available at 10.1186/s12912-022-00940-z.

## Background

The goal of health care is to provide safe and high-quality care for all population [1]. Nurses make up over 50% of the national health workforce in many countries [2, 3]. They provide critical contribution to the people’s health by coordinating and integrating the multiple dimensions of quality of care [4]. Therefore, nurses must be competent in clinical skills and keep up with the updates on technology, knowledge and evidences pertinent to nursing practice. There are many ways to promote the nurses’ ability; and the continuing professional development (CPD) has been considered as one of the important approaches to helping them maintain skills and motivation for work to provide patients safe care [5, 6].

The CPD is defined by American Nurses Association (ANA) as “a lifelong process of active participation by nurses in learning activities that assist in developing and maintaining their continuing competence, enhancing professional practice and supporting achievement of their professional goals” [7]. Nurse’ CPD is fundamental to professionalism and lifelong learning and is considered as a vital factor for updating nurses’ knowledge and skills [8–10]. Previous studies reported that the promoting of CPD for nurses in the clinical context was fundamental, due to its positive effect on patients, professional and the organizations [6, 11]. Specifically, CPD enables nurses to cope with many kinds of public health emergencies, and contributes to improving the quality of care as well as health outcomes [2, 3, 12]. Therefore, it is an important part for nursing human resource management to promote nurses’ CPD, and equip them with the necessary professional knowledge and ability [13, 14].

The global nursing status report in 2020 showed that there was a shortage of 5.9 million nurses across the world [15]. An inadequate supply of nursing staff paired with increasing workloads hamper nurses’ development opportunities [16]. Previous studies identified that the culture and environment of workplace had significant impact on nurses’ development [16, 17]. Besides, demographic differences, particularly age, may influence their participation of development; it has been reported that older nurses were less attracted to pursue additional academic education opportunities for their development when comparing with younger counterparts [18, 19].

Though CPD could contribute to self-improvement, many nurses were confused about what CPD was and what it meant to their profession and their own [20]. By the end of 2020, there were 4.70 million nurses in China, and more than 70% of them have a junior college degree or above. According to the policy enacted by National Health Commission, the organizations should ensure that over 90% clinical nurses will participate in CPD activities by 2025. Moreover, if nurses want to develop as a specialist nurse, they should complete the length of study required [21]. In order to strengthen the professional ability of nurses, the hospital will require nurses to obtain professional learning credits every year to meet the annual performance appraisal standards. Nursing staff and their development level are an important part of the medical health system that could directly affect the quality of caregiving. However, previous study found nurses’ experiences regarding their CPD is a key issue that has not been deeply explored [6]. Given the development of nursing and the high demand for nursing staff, it is necessary to have a better understanding of nurses’ professional development condition and implement appropriate interventions for nurses’ CPD. Therefore, the aim of this study was to explore Chinese nurses’ perceptions and challenges of CPD.

## Methods

### Design

Our research used a qualitative descriptive with semi-structured face-to-face interviews approach to explore Chinese nurses’ perception of CPD. The consolidated criteria for reporting qualitative research (COREQ) checklist was used to ensure the quality of research [22] (see Additional file 1).

### Setting and sample

This qualitative study took place in a university-affiliated public tertiary hospital in the central region of China, with more than 7000 beds and over 4000 nurses. This hospital has implemented magnetic management since 2012, and meanwhile supply various free resources such as online and offline learning for nurses’ continuing learning. Besides, the hospital supplies financial support for the training of specialist nurses every year.

Purposive sampling was used until data reached theoretical saturation (i.e., no new themes were identified). The eligible criteria were having a nurse qualification certificate and participating in clinical nursing work in this hospital. Participants were excluded if they were intern nurses or trainee nurses from other hospitals. Eligible nurses were contacted by one researcher (YL), who introduced the research aims and procedures for participants. If they presented interest, they were asked to participate in the research interview. All recruited participants have CPD experiences.

### Data collection

Based on the aims of the research, a semi-structured interview guide was formed through extensive review of relevant literature, and discussion among the research team members: experiences with professional development status; influences on CPD; the coping strategies used to deal with challenges during CPD; need in the process of CPD. Interviews were conducted by two first authors (XYY and YH), who had no prior contact with respondents. The semi-structured interview outline would be sent to the interviewees by email one day before, so that they can think about relevant issues in advance. All participants signed informed consents and provided their demographic data before interview. Before the formal interview, we pre-interviewed a nurse and adjusted the interview questions according to the interview outcomes. The final interview questions included in the interview guides are presented in Table 1. These interviews were recorded using a mini-recording device in a quiet conversation room in participants’ workplace and lasted from 30 to 45 min. Field notes were taken during and after interviews. The interview data were collected from July to August 2020.

**Table 1:** Semi-structured interview guide

No.	Questions
1	What is your opinion on your professional development?
2	What may influence your continuing professional development?
3	What were your difficulties and challenges during continuing professional development?
4	How do you cope? Who or what helped you during these periods?
5	What supports do you need in the process of continuing professional development?

### Data analysis

We used thematic analysis to scrutinize data [23]. Audio recordings were transcribed verbatim immediately after interviews. Transcripts were repeatedly read against the recordings and notes to capture participants’ exact meanings by the two researchers (XYY and YH). These transcripts were sent to the interviewees for validation. Key lines and condensed meaning units were marked in the text, which were then coded to generate initial codes. Similar codes were clustered to create subcategories and categories, which in turn were grouped into themes. The data analysis was ongoing throughout data collection. Table 2 shows a sample of data analysis. The first author did the coding and the others read a sample of coded interviews to check the coding. All authors discussed the assigned codes multiple times, until consensus was reached. Following data analysis, the extracted themes and quotations were translated into English by the researcher (bilingual in English and Chinese), and then back-translated into Chinese by translator to make sure that their meaning were consistent with the original Chinese transcripts [24].

**Table 2:** An example of data analysis

Meaning units	Primary codes	Sub-theme	Theme
“I didn’t think too much about the future development, because I had just entered ICU for less than one year, so … I should be familiar with the work environment”. (Nurse 4) “I have just been working for one year, and I have no idea about my development yet. Now I just hope to … adapt to the working environment”. (Nurse 10)	Have no good idea about future personal development because have to adapt to new environment	Confusion	Having different development phases
“Now, I not only need to do the clinical nursing work, but also take charge of the teaching tasks of our department. Besides … I have participated in the training of specialist nurses, and I am going to applied for health related-qualification certificate next year”. (Nurse 2) “Now I am an on-job postgraduate student, and I want to get promoted to a higher title later. In addition, I have participated in various activities to enhance my comprehensive ability …” . (Nurse 9)	Try to change and improve ability Keep learning and improve comprehensive ability	Exploration	
“Now I am working as a teaching supervisor in our department, and I feel very confident that I will do better”. (Nurse 1) “Because I am a graduate student, and I would continue to do the nursing research …”. (Nurse 14)	Be confidence about current development situation Toward to own research interest	Maintenance	

### Rigor

The rigor of the qualitative research was assured by the following four criteria: credibility, transferability, confirmability, and dependability [25]. To achieve credibility, the interviews were guided by interview questions based on a comprehensive literature review, and field notes were made during the interviews. To enhance the confirmability, two researchers independently analyzed the data [26]. Then findings were discussed within research team meetings until consensus was achieved. Member checks were also conducted with two individual participants invited to review and comment on interpretive notes via face to face meetings [26]. For the dependability, verbal and non-verbal data were recorded and interpreted. Besides, quotations were also presented to elaborate each theme and subtheme. Lastly, transferability was established through clearly describe the research design, data collection and analysis process [26].

### Ethical consideration

Permission for conducting the study was granted by the hospital. The participants were informed about this study, and they provided written informed consent after it was emphasized that participation was completely voluntary and participants could withdraw from the research at any time. In addition, participants were informed that their identity was not disclosed at any stage when reporting the result. Only the researchers and research team had access to the data in a password-protected computer.

## Results

A total of 14 participants were interviewed, none refused or withdrew. The mean age of the participants was 33, and 11 of the 14 participants were married. The detailed characteristics of the nurses are presented in Table 3.

**Table 3:** Characteristics of the fourteen nurses

No.	Department	Age (years)	Years of experience in nursing	Marital status	Education level	Position	Competency level
1	Operating department	32	10	Married	Master	Educational nurse	N2
2	Surgical department	30	9	Married	Bachelor	Educational nurse	N2
3	Emergency department	30	10	Married	Bachelor	Nurse	N2
4	Internal medical department	26	4	Single	Bachelor	Nurse	N3
5	Internal medical department	31	10	Married	Bachelor	Nurse	N1
6	Pediatric department	32	7	Married	Bachelor	Nurse	N2
7	Surgical department	39	19	Married	Bachelor	Head nurse	–
8	Surgical department	40	17	Married	Bachelor	Specialist nurse	–
9	Gynecology department	28	4	Single	Bachelor	Nurse	N4
10	Rotating nurse	23	2	Single	Bachelor	Nurse	N1
11	Pediatric department	35	14	Married	Master	Head nurse	–
12	Oncology department	38	18	Married	Bachelor	Nurse	N3
13	Geriatric department	35	15	Married	Bachelor	Nurse	N3
14	Internal medical department	38	16	Married	Master	Clinical research nurse	N3

Four themes emerged from the interviews: improving specialty ability, different development phases, the importance of personal effort, the obstacle of work-family conflict. Participant’s quotations were used as exemplars to illustrate the important issues experienced by participants and to support each theme and subtheme.

### Improving specialty ability

A specialist nurse is qualified with a high level and expertise in a particular or specialized area of nursing. When interviewees talked about their CPD, they expected to strengthen the ability of clinical specialty and become specialist nurse. Accordingly, the following narratives were recorded:

“*For nurses, expertise is so important. No matter whether you are going to be … a nurse manager or a nurse educator, you need master relevant professional knowledge, which could increase your confidence*”. (Nurse 1)

“*I want to be more proficient in both nurse management and clinical professional knowledge. Thus … , as a head nurse, I also have participated in many academic meetings to get an opportunity to communicate and upgrade knowledge in recent years*”. (Nurse 7)

Moreover, when nurses get knowledge and ability in a certain specialty area, they may feel valued and respected, as one nurse said: “I thought that the personal professional development needed to be more specialized in the clinical, because … if nurses expected to win respect, no matter whether it came from the doctor or the patient, they must master a lot of knowledge”. (Nurse 3).

### Different development phases

Nurses’ professional development was categorized into three stages including confusion, exploration and maintenance.

#### Confusion

For novice nurses with few years working experience, they may feel confused about their growth and have no idea about where they should go. These nurses have to take some time and energy to be more familiar with workplace environment and clinical practice. The following narrative highlighted these findings:

“*I didn’t think too much about the future development, because I had just entered ICU for less than one year, so … I should be familiar with the work environment*”. (Nurse 4)

“*I have just been working for one year, and I have no idea about my development yet. Now I just hope to … adapt to the working environment*”. (Nurse 10)

#### Exploration

The continuing learning is necessary for nurses who have to cope with an ever-changing healthcare environment. When nurses get integrated into the clinical practice, they master nursing skills and feel confident in performing their clinical tasks. For further development, they may try to increase their comprehensive ability.

“*Now, I not only need to do the clinical nursing work, but also take charge of the teaching tasks of our department. Besides … I have participated in the training of specialist nurses, and I am going to applied for health related-qualification certificate next year*”. (Nurse 2)

“*Now I am an on-job postgraduate student, and I want to get promoted to a higher title later. In addition, I have participated in various activities to enhance my comprehensive ability …”.* (Nurse 9)

#### Maintenance

For nurses who have gotten target and position on their professional development, they have to enhance ability and keep improving. Accordingly, the following narratives were recorded:

“*Now I am working as a teaching supervisor in our department, and I feel very confident that I will do better*”. (Nurse 1)

“*Because I am a graduate student, and I would continue to do the nursing research …”.* (Nurse 14)

“*As a specialist nurse, of course … , I would can make my profession better*”. (Nurse 8)

### The importance of personal effort

Individual initiative and willingness play important role in CPD. Nurses who are willing to engage in CPD will take use of various resource to keep learning and update knowledge.

“*I have attended a lot of online classes now. For example, I have attended online classes via mobile phone on subway, so … I can arrange time reasonably*”. (Nurse 2)

“*I read every day when I come home after work … , it is more important to read books about cardiovascular medicine, except for nursing books*”. (Nurse 5)

“*If you are willing to pursue self-development, you will get the opportunity*”. (Nurse 12).

### The obstacle of work-family conflict

Work-family conflict arises from competing responsibilities to work and family. Professional development requires time and energy. Nurse staff attend CPD activities in their personal time to meet the requirements of the nursing service. When they use their personal time to participate in CPD activities, doing so directly conflicts with their family life and vacations, especially for nurses at the age of childbearing. The following narrative highlighted these findings:

“*My family need me, and two young children need to be taken care of … These things cost me a lot of time and energy*”. (Nurse 3)

“*I have two children. So … I have to spend much more time to take care of them. … sometimes I feel very … helplessness about my own development*”. (Nurse 13)

## Discussion

This study offers insights into Chinese nurses’ CPD status and related influencing factors. Our results found that nurses oriented to specialty and went through different stages of professional development. Besides, their development was influenced by personal effort and the conflict between work and family.

Nurses included in this study were expected to strengthen their professionalism while referring to professional development. Due to the rapidly changing healthcare context, it is necessary for nurses to enhance their knowledge and ability and adapt to the gradual revolution of medical technology and the diversity of health-care needs [27]. The development of clinical nursing specialists becomes a trend. In the Development Plan for National Nursing Career of the China (2021-2025), it stresses the importance of strengthening nursing professionalism [21]. Those nurses with high-level skills in a special area such as emergency nursing are becoming a valuable resource. Studies have found that clinical nursing specialists could improve patients’ quality of life, shorten length of hospital stay and reduce medical expenses [28, 29].

This study found that the development of nurses showed significant staged characteristics. Young nurses would lack professional confidence when they entered into clinical practice at the beginning [30, 31]. This fact placed those novice nurses in a tenuous position and they experienced stressful and challenging during the nursing professional development [31, 32]. However, nurses with more than 10 years working experience have accumulated rich knowledge and proficient professional skills, and hold the highest perception of their own professional management [33]. Nurse leaders can take advantage of clinical ladder plan to provide a training framework for nurses to promote their continued professional development [34, 35].

In our study, nurses had taken the initiative to utilize various resources to pursue self-development. With the considerable changes of nursing, it was necessary for nurses to inspire their own initiative and motivation in clinical practice in order to maintain competency and provide quality patient care [36, 37]. It was reported that a positive workplace culture through adequate resources of time, staffing and administrative support, which played an important role in nurses’ CPD to ensure their continuous growth in their clinical practice [6, 9, 38]. Therefore, to further strengthen the overall development of nursing staff, nurse leaders need to create a positive workplace culture and motivate nurses who perceive the relevance of CPD to their practice and are supported to access learning.

However, the difficulty to achieve work-family balance is the major obstacle of nurses’ CPD in this study, which was similar to previous study [39]. It has been reported that job demands and job control are the two major factors influencing on the work-family balance of nurses [40, 41]. Nurses between 30 and 40 years old are the backbone of the department, and their multiple roles in the family can’t be ignored. Thus, the work-family conflict arises from competing responsibilities to work and their family [41, 42]. Studies have reported that specific family factors can importantly predict nurses’ intention to leave work such as family needs or kinship responsibilities [42, 43]. To alleviate this dilemma, nurse leaders should attach attention to the balance of work-family among nurses in this age group, and take effort to meet them needs, such as proper management of human resources and facilitating their attendance at these learning activities within the workday.

### Implications

Exploring the nurses’ CPD could present evidence for nurse leaders to promote the overall development of nursing staff. Nurse’ CPD is a continuing process throughout their professional career, and it is important to update their knowledge and skills to meet the challenge of nursing development. However, due to their clinical position and age, nurses may possess different goals, motivations and needs to participate in CPD. Therefore, nurse leaders should take nurses’ particular professional situations and their real needs into consideration, and provide support for their access to CPD. Besides, nurse leaders should create a positive workplace culture and provide flexible work practices for nurses to balance between their professional and personal lives.

### Limitations

Several limitations should be noted in this study. First, participants were recruited in one clinical environment and the results may be specific to this institution. Second, the interview data were translated from Chinese to English, it is always a risk to misinterpret and mislay some of the meaning when translating data. Another limitation was that participants were recruited purposively from one institution in China. Therefore, caution should be practiced in the transferring of these findings to other clinical settings.

## Conclusions

The study findings could supply nurse leaders with a more in-depth understanding of nurses’ CPD. Nurses expect to improve their clinical specialty, and their development is characterized by different phases. Personal effort is considered as the main stimulating factor, while work-family conflict is the major obstacle to their development. Thus, it is necessary to strengthen job management for highlighting the value of position, enrich the training of specialist nurses, and implement hierarchical training to promote the development of sustainable workforce in the health care institution. Besides, it is imperative for the nurse leaders to formulate appropriate human resource strategies that could provide a flexible working system to balance work and family for nursing staff, which is conducive to the development of nursing staff and stabilizes the nursing team.

## Supplementary Information

**Additional file 1: Table 1.** COREQ (Consolidated criteria for reporting qualitative research) Checklist.

## Data Availability

Data is available upon reasonable request from the corresponding author.

## Abbreviations

CPD: Continuing professional development
ANA: American Nurses Association
COREQ: Consolidated criteria for reporting qualitative research

## References

[1] International Council of Nurses. International workforce forum calls for urgent action from governments to address global nursing shortage. 2019. Available from:https://www.icn.ch/news/icn-international-workforce-forum-calls-urgentaction-gover nments-addre ss-global-nursing (Accessed January 20 2021).

[2] Pulcini J, Jelic M, Gul R, Loke AY (2010). An international survey on advanced practice nursing education, practice, and regulation. J Nurs Scholarsh.

[3] World Health Organization (2018). Density of nursing and midwifery personnel.

[4] Katsikitis M, McAllister M, Sharman R, Raith L, Faithfull-Byrne A, Priaulx R (2013). Continuing professional development in nursing in Australia: current awareness, practice and future directions. Contemp Nurse.

[5] Steven A, Larkin V, Stewart J, Bateman B (2018). The value of continuing professional development: a realistic evaluation of a multi-disciplinary workshop for health visitors dealing with children with complex needs. Nurse Educ Today.

[6] Vázquez-Calatayud M, Errasti-Ibarrondo B, Choperena A (2020). Nurses' continuing professional development: a systematic literature review. Nurse Educ Pract.

[7] American Nurses Association and National Nursing Staff Development Organization (2010). Nursing professional development: scope and standards of practice.

[8] Kane RL, Shamliyan TA, Mueller C, Duval S, Wilt TJ (2007). The association of registered nurse staffing levels and patient outcomes: systematic review and meta-analysis. Med Care.

[9] King R, Taylor B, Talpur A, Jackson C, Manley K, Ashby N (2021). Factors that optimise the impact of continuing professional development in nursing: a rapid evidence review. Nurse Educ Today.

[10] Mlambo M, Silén C, McGrath C (2021). Lifelong learning and nurses' continuing professional development, a metasynthesis of the literature. BMC Nurs.

[11] Hariyati RTS, Safril S (2018). The relationship between nurses' job satisfaction and continuing professional development. Enferm Clin.

[12] Pool IA, Poell RF, Berings MG, Ten Cate O (2016). Motives and activities for continuing professional development: an exploration of their relationships by integrating literature and interview data. Nurse Educ Today.

[13] Jokiniemi K, Suutarla A, Meretoja R, Kotila J, Axelin A, Flinkman M (2020). Evidence-informed policymaking: modelling nurses' career pathway from registered nurse to advanced practice nurse. Int J Nurs Pract.

[14] Randolph PK, Hinton JE, Hagler D, Mays MZ, Kastenbaum B, Brooks R (2012). Measuring competence: collaboration for safety. J Contin Educ Nurs.

[15] World Health Organization (2020). State of the World’s nursing report.

[16] Coventry TH, Maslin-Prothero SE, Smith G (2015). Organizational impact of nurse supply and workload on nurses continuing professional development opportunities: an integrative review. J Adv Nurs.

[17] Io M. The future of nursing: leading change, advancing. Health. 2010; http://thefutureofnursing.org/IOM-Report on 22 May 2014.

[18] Bell JA (2013). Five generations in the nursing workforce: implications for nursing professional development. J Nurses Prof Dev.

[19] Pool I, Poell R, ten Cate O (2013). Nurses' and managers' perceptions of continuing professional development for older and younger nurses: a focus group study. Int J Nurs Stud.

[20] Horn K, Pilkington L, Hooten P (2019). Pediatric staff nurses' conceptualizations of professional development. J Pediatr Nurs.

[21] National Health Commission. Development plan for national nursing career of the PRC (2021-2025). http://www.nhc.gov.cn/yzygj/s7653pd/202205/ 441f75ad347b4ed68a7d2f2972f78e67.shtml (Accessed May13 2022).

[22] Tong A, Sainsbury P, Craig J (2007). Consolidated criteria for reporting qualitative research (COREQ): a 32-item checklist for interviews and focus groups. Int J Qual Health Care.

[23] Miles MB, Huberman, AM, Saldaña J. Qualitative data analysis: A methods sourcebook (3rd ed.) Los Angeles, CA: S; 2014.sourcebook (3rd ed.) Los Angeles, CA: S; 2014.

[24] Chen HY, Boore JR (2010). Translation and back-translation in qualitative nursing research: methodological review. J Clin Nurs.

[25] Speziale HS, Streubert HJ, Carpenter DR. Qualitative research in nursing: advancing the humanistic imperative. Lippincott Williams & Wilkins. 2011.

[26] Polit DF, Beck CT (2016). Nursing research: generating and assessing evidence for nursing practice.

[27] Kavanagh JM, Szweda C (2017). A crisis in competency: the strategic and ethical imperative to assessing new graduate nurses' clinical reasoning. Nurs Educ Perspect.

[28] Comiskey C, Coyne I, Lalor J, Begley C (2014). A national cross-sectional study measuring predictors for improved service user outcomes across clinical nurse or midwife specialist, advanced nurse practitioner and control sites. J Adv Nurs.

[29] Cooper MA, McDowell J, Raeside L (2019). The similarities and differences between advanced nurse practitioners and clinical nurse specialists. Br J Nurs.

[30] Henderson A, Eaton E (2013). Assisting nurses to facilitate student and new graduate learning in practice settings: what 'support' do nurses at the bedside need?. Nurse Educ Pract.

[31] Ortiz J (2016). New graduate nurses' experiences about lack of professional confidence. Nurse Educ Pract.

[32] Rudman A, Gustavsson P, Hultell D (2014). A prospective study of nurses’ intentions to leave the profession during their first five years of practice in Sweden. Int J Nurs Stud.

[33] Du L, Mao HB (2020). Perceived organizational career management among nurses working at tertiary hospitals in Hubei province: the influencing factors. J Nurs Sci.

[34] Bitanga ME, Austria M (2013). Climbing the clinical ladder--one rung at a time. Nurs Manag.

[35] Kukkonen P, Leino-Kilpi H, Koskinen S, Salminen L, Strandell-Laine C (2020). Nurse managers' perceptions of the competence of newly graduated nurses: a scoping review. J Nurs Manag.

[36] Munro KM (2008). Continuing professional development and the charity paradigm: interrelated individual, collective and organisational issues about continuing professional development. Nurse Educ Today.

[37] Price S, Reichert C (2017). The importance of continuing professional development to career satisfaction and patient care: meeting the needs of novice to mid-to late-career nurses throughout their career span. Admin Sci.

[38] Falguera CC, De Los Santos JAA, Galabay JR, Firmo CN, Tsaras K, Rosales RA (2021). Relationship between nurse practice environment and work outcomes: a survey study in the Philippines. Int J Nurs Pract.

[39] Pool IA, Poell RF, Berings MG, ten Cate O (2015). Strategies for continuing professional development among younger, middle-aged, and older nurses: a biographical approach. Int J Nurs Stud.

[40] Ng LP, Chen IC, Ng HF, Lin BY, Kuar LS (2017). Influence of job demands and job control on work-life balance among Taiwanese nurses. J Nurs Manag.

[41] Yamaguchi Y, Inoue T, Harada H, Oike M (2016). Job control, work-family balance and nurses' intention to leave their profession and organization: a comparative cross-sectional survey. Int J Nurs Stud.

[42] Labrague LJ, Ballad CA, Fronda DC. Predictors and outcomes of work-family conflict among nurses. Int Nurs Rev. 2020. 10.1111/inr.12642. 10.1111/inr.12642 33165960

[43] Hayes LJ, O'Brien-Pallas L, Duffield C, Shamian J, Buchan J, Hughes F (2006). Nurse turnover: a literature review. Int J Nurs Stud.

